# Efficacy of polymyxin B against different pathogens in carbapenem-resistant gram-negative bacterial pneumonia and its relationship with alveolar epithelial lining fluid concentration: a prospective cohort study

**DOI:** 10.1080/07853890.2026.2752633

**Published:** 2026-11-04

**Authors:** Xuanxi Huang, Mengjie Song, Xueyong Li, Qiong Wu, Wanru Li, Hongqiang Qiu, Wenwei Wu, Hui Zhang, Yu Cheng, Lili Zhou

**Affiliations:** ^a^ Department of Critical Care Medicine, Fujian Medical University Union Hospital, Fuzhou, P.R. China; ^b^ Department of Epidemiology and Health Statistics, School of Public Health, Fujian Medical University, Fuzhou, P.R. China; ^c^ Department of Pharmacy, Fujian Medical University Union Hospital, Fuzhou, P.R. China

**Keywords:** Carbapenem-resistant gram-negative bacteria, pneumonia, polymyxin B, epithelial lining fluid concentration, inhaled therapy, clinical efficacy

## Abstract

**Background:**

The relationship between polymyxin B (PMB) concentrations in alveolar epithelial lining fluid (ELF) and pathogen clearance in carbapenem-resistant Gram-negative bacilli (CRGNB) pneumonia remains unclear. This study evaluated the efficacy of combined nebulized and intravenous PMB and its association with ELF exposure.

**Methods:**

This prospective cohort study included 104 patients with CRGNB pneumonia caused by *Pseudomonas aeruginosa* (CRPA, n=34), *Acinetobacter baumannii* (CRAB, n=38), and *Klebsiella pneumoniae* (CRKP, n=32) receiving nebulized PMB (25 mg every 12 h) plus intravenous PMB (1.25 mg/kg every 12 h) for approximately 14 days. PMB ELF concentrations were measured by the urea dilution method. The primary outcome was bacterial clearance rate; secondary outcomes included time to bacterial clearance, clinical efficacy, fever normalization, 28-day survival, CRGNB-related mortality, inflammatory markers and adverse events.

**Results:**

Bacterial clearance rates (70.59% CRPA, 71.05% CRAB, 78.12% CRKP) and clinical efficacy rates (70.59%, 81.58%, 78.12%) showed no significant intergroup differences (all *p* > .05). CRKP had a shorter time to clearance than CRAB (median, 5.00 vs. 7.00 days, *p* = .004). No significant differences were observed in 28-day survival, CRGNB-related mortality, or adverse events. PMB ELF trough concentration and mechanical ventilation duration were independent predictors of bacterial clearance (both *p* < .05). Higher PMB ELF peak and trough concentrations were associated with shorter clearance/replacement time (both *p* < .05).

**Conclusion:**

The combined PMB regimen achieves favorable bacterial clearance and clinical efficacy in CRGNB pneumonia. Higher local pulmonary PMB exposure was associated with improved bacteriological outcomes, supporting ELF-guided dose optimization.

## 1. Background

The widespread clinical use of antimicrobial agents has been accompanied by the continued emergence and global spread of drug-resistant strains, posing an urgent public health challenge worldwide [1]. Carbapenem-resistant gram-negative bacteria (CRGNB), including carbapenem-resistant *Pseudomonas aeruginosa* (CRPA), *Klebsiella pneumoniae* (CRKP) and *Acinetobacter baumannii* (CRAB), are common causes of ventilator-associated pneumonia (VAP) in intensive care units (ICUs) [2]. Carbapenem resistance in these pathogens substantially limits the availability of effective antimicrobial options and is associated with increased healthcare costs, prolonged hospitalization and higher mortality risk [3]. Treatment options for CRGNB are very limited. The polymyxins used in humans comprise two types, polymyxin B (PMB) and polymyxin E (colistin). PMB is administered as the active drug PMB sulphate, which exerts direct antibacterial effects; colistin, in contrast, is marketed as its prodrug colistin methanesulphonate (CMS), which must be hydrolysed in the kidneys to its active form. PMB achieves higher systemic exposure [4,5], whereas the conversion efficiency of CMS is significantly influenced by renal function [6]. Consequently, owing to its low clinical resistance rate and unique mechanism of action, PMB is regarded as the ‘last line of defense’ against CRGNB infections [7]. However, its use is limited by clinically relevant adverse effects, particularly nephrotoxicity and neurotoxicity during systemic administration [5,8]. Inhaled PMB may additionally cause airway irritation or bronchospasm, while skin hyperpigmentation has also been reported during PMB therapy [5,8,9]. Therefore, treatment strategies that optimize pulmonary exposure while minimizing systemic toxicity are clinically important.

When PMB is administered intravenously to treat CRGNB pneumonia, it binds to the phosphate moiety of lipopolysaccharide (LPS) in the outer membrane of gram-negative bacteria. Through a mechanism known as ‘spontaneous uptake’, it disrupts the integrity of the cell membrane and increases its permeability, leading to leakage of cellular contents and bacterial death [10,11]. However, its high molecular weight results in poor pulmonary permeability, resulting in relatively low drug concentrations in the alveolar epithelial lining fluid (ELF) [12–14]. Our previous work demonstrated that the PMB concentration in ELF is positively correlated with clinical efficacy and serves as an independent predictor of therapeutic outcomes [15]. In that study, patients received nebulized PMB at 25 mg every 12 h, together with intravenous PMB (a loading dose of 2.0 mg/kg followed by 1.25 mg/kg every 12 h). This combined regimen resulted in significantly higher PMB exposure in ELF and was associated with improved clinical outcomes, without a corresponding increase in adverse events [15]. Thus, increasing the concentration of PMB ELF in the affected lung segment plays a crucial role in patient clinical outcomes.

As a method of drug delivery, nebulization transforms medication into a mist that acts directly on the lungs. For polymyxins, particularly in the treatment of CRGNB pneumonia, nebulized administration has attracted attention because it can increase local pulmonary drug exposure while potentially reducing systemic exposure-related toxicity [16,17]. This is particularly relevant given that PMB-related nephrotoxicity remains a significant clinical concern, with the reported incidence of acute kidney injury (AKI) ranging from 10% to 60% across various studies [8]. A systematic review and Bayesian network meta-analysis demonstrated that, in patients with severe CRAB pneumonia, nebulized PMB combined with intravenous infusion showed significantly superior efficacy compared with intravenous administration alone [18]. Additionally, a multicentre matched case-control study concluded that nebulized combined intravenous infusion yielded better clinical outcomes than intravenous infusion alone for CRGNB ventilator-associated pneumonia in critically ill patients [19]. A previous prospective randomized controlled study from our group demonstrated that nebulized PMB exhibited a significant advantage in increasing drug concentration in ELF, with a lower incidence of nephrotoxicity compared with intravenous administration [20]. Furthermore, a population pharmacokinetic model based on 76 patients with CRGNB pneumonia who received nebulized PMB alone or in combination with intravenous administration revealed that nebulization therapy significantly improved ELF concentrations and the probability of achieving therapeutic targets, supporting the pharmacokinetic advantage of the nebulized route [21], consistent with the findings of Ding et al. [22]. In these clinical and pharmacokinetic studies, nebulized PMB was generally administered at 25–50 mg every 12 h, while intravenous PMB commonly included a loading dose of approximately 2.0–2.5 mg/kg followed by maintenance doses of 1.25–1.5 mg/kg every 12 h [19–22]. Consequently, nebulized PMB demonstrates a favourable risk-benefit ratio in the treatment of CRGNB pneumonia and may serve as an effective therapeutic option for this condition.

Although several studies have demonstrated that the combination of nebulized and intravenous PMB may be associated with improved cure rates in patients with CRGNB pneumonia [23], the baseline susceptibility to PMB, specifically the distribution of minimum inhibitory concentration (MIC) values, may vary among CRPA, CRKP and CRAB [24], thereby affecting its therapeutic efficacy [25]. Furthermore, CRPA and CRAB exhibit a greater propensity for biofilm formation, which significantly restricts the penetration and bactericidal activity of PMB against sequestered bacteria [26]. This often results in incomplete bacterial eradication and an increased risk of clinical recurrence [27]. Although CRKP can also form biofilms, its mechanisms of polymyxin resistance, such as lipopolysaccharide modification, may be more common, leading to failure of initial bactericidal activity [28,29]. Additionally, infection-induced local inflammation and secretion properties (e.g. the copious purulent sputum typical of CRPA) can hinder drug-bacteria contact. Thus, the required threshold for an effective ELF concentration may vary by bacterial species, highlighting the need for pathogen-specific, individualized treatment regimens [30]. The correlation between ELF concentrations of PMB and the eradication of different pathogens has yet to be elucidated. Therefore, the primary aim of this prospective cohort study was to evaluate the bacteriological efficacy of combined nebulized and intravenous PMB in patients with CRGNB pneumonia caused by different pathogens, including CRPA, CRAB and CRKP. We also compared clinical outcomes and safety profiles across pathogen groups and investigated the association between PMB concentrations in ELF and bacterial clearance. By clarifying the relationship between local pulmonary PMB exposure and pathogen eradication, this study aimed to provide evidence for optimizing individualized PMB dosing regimens in CRGNB pneumonia.

## 2. Materials and methods

### 2.1. Study design and patient population

This prospective cohort analysis was derived from the database of a parent randomized controlled trial conducted in the ICU of Fujian Medical University Union Hospital between January 2022 and March 2026 (ChiCTR2100044087). Consecutive patients were screened according to the eligibility criteria of the present analysis, without selective or quota sampling. During this period, approximately 250 patients with CRGNB pneumonia were identified in our ICU; CRAB accounted for approximately 40%, whereas CRPA and CRKP each accounted for approximately 30%. After applying strict inclusion, exclusion and withdrawal criteria, 104 eligible patients were included in the final analysis. Additional variables, including bacterial clearance/replacement time and concomitant antibiotic regimens, were extracted from electronic medical records before analysis, and the hypotheses were specified before data extraction. The study protocol was approved by the Ethics Committee of Fujian Medical University Union Hospital (No. 2020KY0136/2022YF033-03). As eligible patients with CRGNB pneumonia were usually critically ill, had artificial airways and required mechanical ventilation, written informed consent was obtained from legally authorized representatives, usually immediate family members such as spouses, children, or parents, after they had been fully informed of the study purpose, procedures, potential risks and benefits. The study was conducted in accordance with the Declaration of Helsinki.

The original randomized controlled trial was designed as a non-inferiority study. Accordingly, the sample size was calculated using a non-inferiority framework with a pre-specified non-inferiority margin of 15%, a one-sided significance level (α) of 0.017 and a statistical power of 92%. The non-inferiority margin was determined based on previous published evidence, based on a published meta-analysis [31] and clinical judgement. The present study represents a post hoc analysis of the original trial and was not designed to formally test non-inferiority. Therefore, all statistical analyses in the current study were performed using conventional superiority-based methods appropriate for the objectives of this post hoc analysis.

Patients were included in this study if they met the following inclusion criteria: (1) diagnosis of CRGNB pneumonia according to the Chinese Thoracic Society guidelines [32], defined by both clinical and microbiological evidence: new or progressive pulmonary infiltrates on chest radiography or computed tomography, accompanied by at least two of the following – fever >38 °C or hypothermia <36 °C, leukocytosis or leukopoenia and purulent airway secretions; and isolation of CRPA, CRAB, or CRKP from respiratory specimens obtained by fibreoptic bronchoscopy; (2) bacterial culture of sputum yielding a single strain of CRGNB that was susceptible to PMB *in vitro*; (3) receipt of nebulized combined with intravenous PMB for at least three days; (4) presence of an artificial airway. Exclusion criteria were as follows: (1) judged to be imminently dying; (2) presence of severe hepatic insufficiency (Child-Pugh class C) or renal insufficiency (creatinine clearance < 30 mL/min); (3) evidence of gram-positive bacterial, viral, or fungal infections, or infections outside the lungs; (4) incomplete clinical data; (5) cases in which colonization could not be clearly distinguished from infection. Withdrawal criteria included: (1) voluntary request to withdraw from the study; (2) occurrence of significant fluctuations in heart rate or blood pressure, or a marked decrease in pulse oxygen saturation, during real-time electrocardiograph (ECG), blood pressure and oxygen saturation monitoring; (3) development of severe neurological or renal adverse reactions associated with PMB; (4) patients who developed opportunistic fungal infection during the study period were withdrawn from the analysis.

All participants received combined nebulization and intravenous infusion for approximately 14 days. An intravenous loading dose of 2.0 mg/kg of PMB was given first, followed by maintenance doses of 1.25 mg/kg every 12 h, each infused over approximately 1 h. Nebulized PMB was administered at a dose of 25 mg every 12 h using a vibrating mesh nebulizer, with intravenous and nebulized administration occurring simultaneously. The PMB dosing regimen was determined according to the Chinese multidisciplinary expert consensus [9] on the rational clinical use of polymyxins and was approved by the Pharmacy Administration Committee of Fujian Medical University Union Hospital. Therefore, the selected intravenous and nebulized doses were considered clinically appropriate and consistent with local institutional practice. Prior to nebulization, sputum was cleared from the airways, and bronchodilation was induced with a β2-agonist approximately 20 min beforehand. No other nebulized medications were co-administered during or immediately following PMB nebulization. A 25 mg dose of PMB was dissolved in 5 mL of sterile water for injection to prepare the nebulization solution. The solution was administered every 12 h *via* a vibrating mesh nebulizer placed 15 cm proximal to the Y-connector in the ventilator inspiratory circuit, for 20–30 min or until the nebulizer chamber was empty. Mechanical ventilation was set to synchronized intermittent mandatory ventilation (SIMV)+ pressure support ventilation (PSV) mode, with a tidal volume of 8 mL/kg, respiratory rate to 18 breaths/min and an inspiratory-to-expiratory ratio of 1:2. The inspiratory pause was set to 20% of the inspiratory time. Positive end-expiratory pressure (PEEP) was titrated to achieve optimal oxygenation (5–8 cmH_2_O). The humidification system was turned off during nebulization to prevent medication from settling in the ventilator tubing, which would result in drug waste. The ventilator exhalation filter was replaced after each nebulization session to prevent medication from escaping into the ambient air, which could contribute to drug resistance.

### 2.2. Sampling and data collection

After at least four doses of PMB had been administered, blood samples (3 mL) were collected in EDTA tubes, and bronchoalveolar lavage fluid (BALF) was collected at the end of the fifth infusion or nebulization (peak concentration, *C*_peak_) and at 12 h after the start of the fifth dose (trough concentration, *C*_trough_). These sampling time points were predefined as protocol-specified steady-state peak and trough concentrations. The acceptable time window for each sampling point was within ±10 min of the scheduled time. Bronchoalveolar lavage was performed using a flexible fibreoptic bronchoscope in accordance with standard operating procedures.

A preoperative chest computerized tomography (CT) scan was performed, and the images were carefully reviewed to determine the location of the lesion. For patients with bilateral diffuse lesions, the right middle lobe was selected as the preferred site for lavage. For localized lesions, bronchoalveolar lavage was performed at the site of the most severe lesion (e.g. where CT images showed ground-glass opacities or consolidation). During standard flexible bronchoscopy, the distal end of the bronchoscope was positioned within the selected bronchopulmonary segment, and lavage was performed at the same location using normal saline.

The first 20 ml of lavage fluid was used to dislodge sputum plugs from the small airways and is discarded; the second 20 mL was used to collect approximately 6 mL of BALF [33]. BALF samples were considered acceptable for ELF concentration estimation only when the lavage recovery rate was ≥30% [34], no patients were excluded because of insufficient BALF recovery. Blood and BALF samples were centrifuged (4 °C, 3000 rpm, 10 min). The supernatant plasma and BALF samples were stored at −80 °C for further analysis. This sample-processing procedure was consistent with our previously validated LC-MS/MS method for PMB quantification, in which PMB stability during sample preparation and storage was confirmed [21,35].

Additional information was derived from electronic medical records, including demographic data, clinical data (primary diagnosis, microbiological diagnosis of CRGNB pneumonia, comorbidities, Acute Physiology and Chronic Health Evaluation (APACHE II) score, Sequential Organ Failure Assessment (SOFA) score, septic shock status, duration of mechanical ventilation, clinical outcomes, microbiological results, mortality from CRGNB-associated pneumonia and 28-day survival rate), laboratory tests (including inflammatory markers, body temperature, pathogen identification and antimicrobial susceptibility testing), PMB administration details, concurrent use of other antibiotics within seven days prior to PMB initiation, glucocorticoid therapy within 28 days prior to PMB initiation and drug-related adverse effects.

### 2.3. Bioanalysis

PMB concentrations in plasma and BALF were measured using an ultra-fast liquid chromatography (Jasper^™^)-tandem mass spectrometry (AB SCIEX Triple Quad^™^ 4500MD) system, according to the method described in our previous publication [35]. Specifically, protein precipitation was performed by adding 0.1% formic acid in acetonitrile. Samples were then separated on a Shim-pack GSP-HPLC C_18_ column and detected within 4 min using positive-ion mass spectrometry with multiple reaction monitoring.

PMB concentrations in ELF were derived from BALF concentrations using the urea dilution method described in the literature [36,37]. The calculation was performed as follows: C_PMB ELF_ = C_PMB BALF_ × urea dilution index (urea plasma/urea BALF). Plasma and BALF urea levels were quantified using an AU 5800 automated biochemical analyser (Beckman Coulter, Indianapolis, IN, USA).

Respiratory isolates were identified using standard microbiological procedures and an automated microbial identification system. Routine antimicrobial susceptibility testing was performed according to institutional laboratory protocols. PMB MICs were determined by broth microdilution, the recommended method for polymyxin susceptibility testing and interpreted according to current CLSI and/or EUCAST criteria [38,39]. Quality control was performed using standard reference strains, including *Escherichia coli* ATCC 25922 and *Pseudomonas aeruginosa* ATCC 27853.

### 2.4. Outcome measures

The primary outcome was the bacterial clearance rate, assessed at the end of the treatment course. From the first dose of nebulized PMB, respiratory specimens were collected for culture every other day, and eradication or replacement was confirmed when three consecutive cultures obtained at two-day intervals met the predefined microbiological criteria. Secondary outcomes included bacterial clearance time, clinical efficacy rate, time to temperature normalization, 28-day survival rate and CRGNB-related mortality (observed over a 28-day period starting from the initial PMB dose). Additionally, changes in inflammatory markers – including procalcitonin (PCT), C-reactive protein (CRP) and interleukin-6 (IL-6) – were evaluated before and after PMB treatment. The incidence of adverse events, such as bronchospasm, nephrotoxicity and skin hyperpigmentation, was also recorded. Furthermore, predictive factors for favourable bacterial clearance were identified, and the correlation between PMB ELF *C*_peak_ and *C*_trough_ and bacterial clearance time was analysed.

Microbiological outcomes primarily included bacterial eradication, defined as the confirmed absence of the original pathogen in three consecutive sputum cultures. Bacterial replacement was defined as the consecutive isolation of a different pathogen on three instances following the disappearance of the original pathogen. Bacterial persistence (non-clearance) was defined as the continued presence of the original pathogen in sputum cultures, accompanied by persistent or worsening clinical signs and symptoms of infection. The total bacterial clearance rate (%) was calculated as follows: (number of cases with bacterial eradication + number of cases with bacterial replacement)/total number of cases × 100%. This rate was determined based on the primary pathogenic bacteria. Colonizing bacteria (i.e. different types of CRGNB) were excluded from the analysis. Bacterial clearance/replacement time was defined as the duration from the start of treatment to the first time point at which three consecutive sputum cultures, collected at two-day intervals, showed either disappearance of the original pathogen or replacement of the original pathogen by another pathogen.

Clinical outcomes were categorized as clinical cure, clinical improvement, or clinical failure. Clinical cure was defined as the complete resolution of infection-related clinical symptoms, signs, aetiological findings, CT imaging findings and laboratory results to normal or pre-infection baseline at the end of PMB therapy. Clinical improvement was defined as partial resolution or improvement of the aforementioned parameters at the end of PMB therapy compared with baseline. Clinical failure was defined as no significant improvement or deterioration of these parameters, or the necessity to switch to alternative therapeutic regimens after PMB treatment. Clinical cure and clinical improvement were considered favourable clinical outcomes, whereas clinical failure was considered an unfavourable clinical outcome [14]. The clinical efficacy rate (%) was calculated using the following formula: ([number of patients with clinical cure + number of patients with clinical improvement]/total number of patients) × 100%. Data were independently evaluated by two physicians who were blinded to group assignments. Any discrepancies were resolved by consulting a third investigator to reach consensus.

The time to temperature normalization was defined as the time from enrolment to fever resolution (body temperature ≤ 37.2 °C sustained for ≥ 24 h). Survival rates were evaluated over a maximum follow-up period of 28 days. CRGNB-related death was defined as mortality closely associated with CRGNB pneumonia, respiratory failure, or septic shock [40].

Nephrotoxicity was defined as the occurrence of AKI according to the Kidney Disease: Improving Global Outcomes (KDIGO) criteria [41]. Specifically, AKI was defined as an increase in serum creatinine (SCr) of ≥0.3 mg/dL within 48 h, or an increase in SCr to 1.5–1.9 times baseline (Stage 1), 2.0–2.9 times baseline (Stage 2), or ≥3.0 times baseline (Stage 3) within seven days, or urine output <0.5 mL/kg/h for 6 h. Skin hyperpigmentation was evaluated by investigators utilizing the Fitzpatrick scale [42]. For inhalation therapy, bronchospasm was identified if patients developed tachypnoea, coughing, dyspnoea, or dry rales secondary to nebulization-induced airway irritation during the procedure.

### 2.5. Statistical analysis

Statistical analyses were performed using SPSS version 25.0. Analyses were conducted using complete cases with available baseline, microbiological, pharmacokinetic, treatment and clinical outcome data, and no imputation was performed. Normally distributed continuous variables were presented as mean ± standard deviation (SD), whereas non-normally distributed variables were presented as medians and interquartile range (IQR). Intergroup comparisons were performed using one-way ANOVA for normally distributed data and the Kruskal-Wallis H test for non-normally distributed data. Post hoc pairwise comparisons were performed using the Mann-Whitney U test with Bonferroni correction. Categorical data were expressed as frequencies and percentages and analysed using the Pearson chi-square test or Fisher’s exact test, as appropriate. Binary logistic regression was employed to identify independent predictors of favourable bacterial clearance. Multiple linear regression was used to evaluate the correlation between *C*_peak_ and *C*_trough_ of PMB in ELF and the time to bacterial clearance. Statistical significance was defined as *p* < .05.

## 3. Results

### 3.1. Patient demographics and baseline clinical characteristics

Sample size estimation was performed using PASS 2021 (version 21.0.9, NCSS, LLC, Kaysville, UT, USA), with bacterial clearance rate as the primary endpoint. Based on the sample size calculation for non-inferiority testing (significance level α = 0.017), a total of 104 patients were enrolled, comprising 34 in the CRPA group, 38 in the CRAB group and 32 in the CRKP group. This sample size yielded a statistical power of 0.921, confirming that the study was adequately powered to detect the predefined differences.

Based on the statistical analysis (Table 1), there were no significant intergroup differences in baseline characteristics, including age, sex, aetiology of ICU admission, SOFA and APACHE-II scores, concomitant septic shock, duration of PMB therapy, mean PMB plasma concentrations, PMB ELF peak and trough levels, history of corticosteroid co-administration, or duration of mechanical ventilation (all *p* > .05). Comparison of comorbidities revealed that diabetes mellitus was significantly more prevalent in the CRPA group (*p* < .05); however, no significant differences were observed for other comorbidities among the groups (*p* > .05). Notably, CRAB exhibited the highest sensitivity to PMB, with MIC values significantly lower than those in the CRPA group (*p* < .001). After Bonferroni correction, no statistically significant differences in PMB MICs were observed between the CRAB and CRKP groups (*p* = .025) or between the CRPA and CRKP groups (*p* = .075). Significant intergroup differences were observed in the use of combination therapy (*p* < .001). Guided by the antimicrobial susceptibility profiles of the isolates, piperacillin-tazobactam was predominantly used in combination in the CRPA group, whereas tigecycline was more frequently co-administered in the CRAB and CRKP groups (Figure 1).

**Figure 1.**
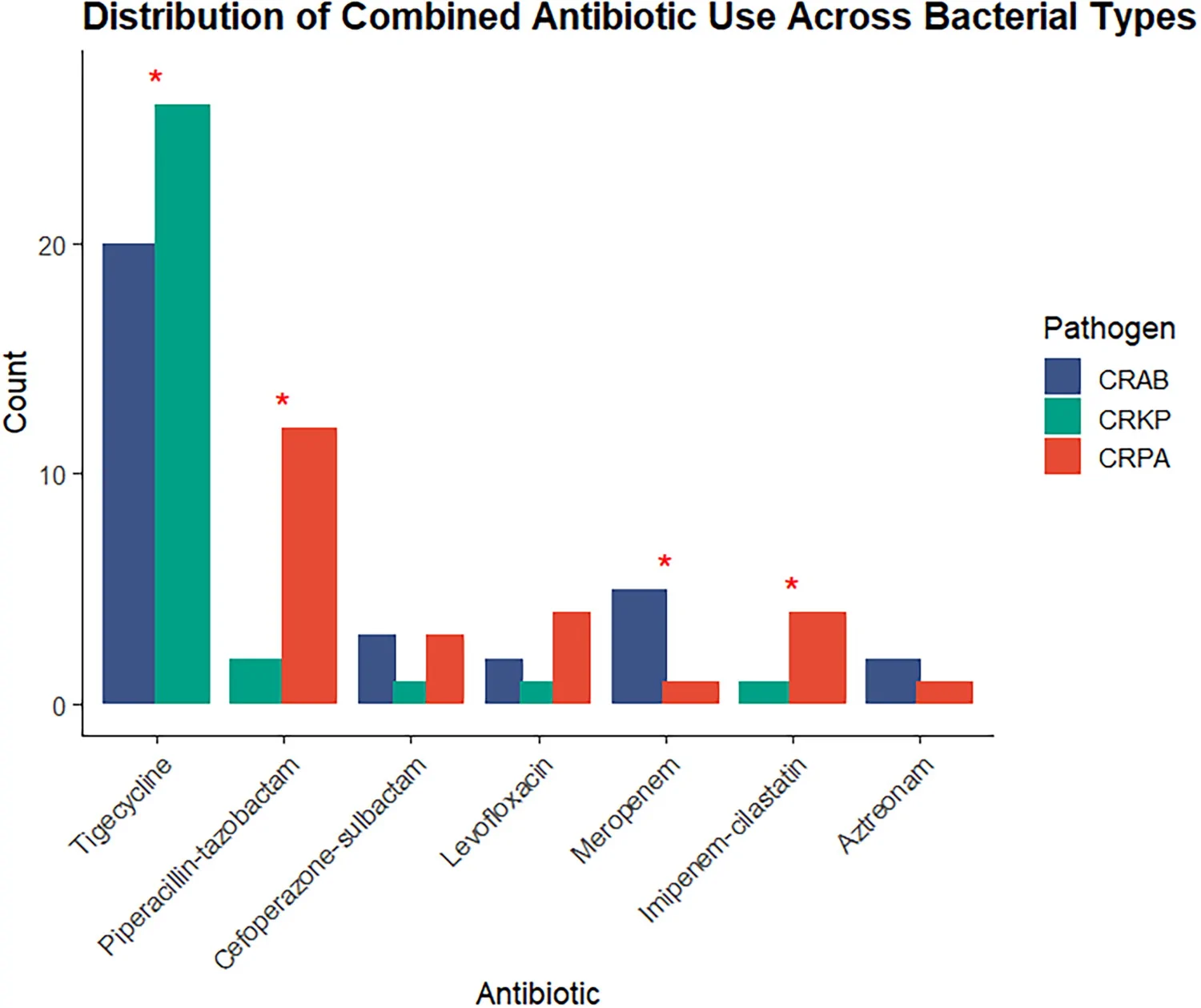
Distribution of combination antibiotic therapy across bacterial types (CRAB, CRKP and CRPA). Bars represent the number of patients receiving each antibiotic within each bacterial group. Asterisks (*) denote statistically significant differences in the distribution of antibiotic use across bacterial types (*p* < .05).

**Table 1.** Comparison of clinical characteristics among three groups of patients infected with different pathogens.

Variable	CRPA (*n* = 34)	CRAB (*n* = 38)	CRKP (*n* = 32)	*p* Value
Age (years)	65.41 ± 13.27	66.39 ± 13.98	61.53 ± 19.00	.398
Gender (*n*, %)				
Male	31 (91.18%)	27(71.10%)	24 (75.00%)	.092
Female	3 (8.82%)	11(28.90%)	8 (25.00%)	
Reason for ICU admission (*n*, %)				.092
Acute respiratory failure	29 (85.29%)	34 (91.90%)	27 (84.38%)	
Multiple trauma	0 (0.00%)	2 (5.40%)	4 (12.50%)	.569
Postoperative	8 (23.53%)	5(13.50%)	5 (15.63%)	.094
Comorbidities (*n*, %)				
Cardiovascular diseases	20 (58.82%)	17 (45.90%)	12 (37.50%)	.215
Cerebrovascular diseases	9 (26.47%)	14(37.80%)	10 (31.25%)	.587
Chronic pulmonary diseases	3 (8.82%)	5 (13.50%)	1 (3.13%)	.381
Chronic liver diseases	1 (2.94%)	0(0.00%)	1 (3.13%)	.535
Diabetes	13 (38.24%)	5 (13.50%)	4 (12.50%)	.020*
Chronic kidney disease	6 (17.65%)	3(8.10%)	1 (3.13%)	.119
Solid tumour	9 (26.47%)	8 (21.60%)	11 (34.38%)	.491
Hematologic malignancy	0 (0.00%)	3 (8.10%)	3 (9.38%)	.211
SOFA score (median, range)	6.00 (4.00, 8.00)	6.00 (4.00, 7.75)	8.00 (4.75, 10.00)	.109
APACHE-II score (median, range)	18.00 (14.25, 25.75)	17.00 (13.25, 25.75)	20.00 (15.75, 27.00)	.416
Sepsis shock (*n*, %)	22 (64.71%)	21(55.30%)	24 (75.00%)	.228
PMB usage duration (days)	14.00 (9.25, 18.75)	12.50 (10.00, 14.00)	13.50 (11.00, 15.00)	.340
Minimum inhibitory concentration of PMB	1.50 (0.50, 2.00)	0.50 (0.50, 0.88)	0.75 (0.50, 2.00)	<.001*
Mean PMB blood concentration (mg/L)	2.31 (2.04, 2.76)	2.40(2.09, 2.65)	2.29 (2.03, 2.79)	.980
PMB ELF peak concentration (mg/L)	11.58 (6.06, 16.30)	11.08 (4.32, 35.06)	9.95 (4.31, 51.73)	.534
PMB ELF trough concentration (mg/L)	1.49 (0.96,3.37)	1.73 (0.71, 3.49)	1.39 (0.64, 3.88)	.953
History of glucocorticoid use (n)	5 (14.71%)	4 (10.50%)	9 (28.13%)	.135
Duration of mechanical ventilation (days)	10.00 (7.00,14.75)	12.50 (7.25, 20.75)	10.50 (9.00, 13.25)	.430

* *p* < .05 indicates statistically significant differences.

### 3.2. Clinical efficacy and safety profile of PMB for pneumonia across different pathogens

The overall bacterial clearance rates for the CRPA, CRAB and CRKP groups were 70.59%, 71.05% and 78.12%, respectively, with no significant intergroup differences (*p* > .05). In contrast, bacterial clearance/replacement time differed significantly among the three groups (*p* < .05). Further pairwise comparisons indicated that, while no significant differences existed between the CRPA and CRAB group (*p* = .255) or between the CRPA and CRKP group (*p* = .173), the CRKP group achieved bacterial clearance/replacement significantly faster than the CRAB group (median [IQR]: 5.00 [3.00, 6.00] *vs.* 7.00 [5.00, 12.00] days, *p* = .004) (Table 2). The overall clinical efficacy rates for the CRPA, CRAB and CRKP groups were 70.59%, 81.58% and 78.12%, respectively, with no significant intergroup differences (*p* > .05, Table 3).

**Table 2.** Comparison of bacteriological outcomes and bacterial clearance/replacement time among three groups of patients infected with different pathogens.

Outcome	CRPA (*n* = 34)	CRAB (*n* = 38)	CRKP (*n* = 32)	*p* Value
Clearance (*n*, %)	21 (61.77%)	23 (60.52%)	17 (53.12%)	.366
Replacement (*n*, %)	3 (8.82%)	4 (10.53%)	8 (25.00%)	
Not cleared (*n*, %)	10 (29.41%)	11 (28.95%)	7 (21.88%)	
Total clearance cases (*n*, %)	24 (70.59%)	27 (71.05%)	25 (78.12%)	
Bacterial clearance/replacement time (days)	7.00 (3.25, 10.00)	7.00 (5.00, 12.00)	5.00 (3.00, 6.00)	.021*

*Notes:* Total clearance cases (*n*) = clearance cases (*n*)+ replacement cases (*n*). Total clearance rate = [total clearance cases (*n*)/total cases (*n*)] × 100%.

* *p* < .05 indicates statistically significant differences.

**Table 3.** Comparison of clinical efficacy rates among three groups of patients infected with different pathogens.

Outcome	CRPA (*n* = 34)	CRAB (*n* = 38)	CRKP (*n* = 32)	*p* Value
Cured (*n*)	1 (2.94%)	1 (2.63%)	0 (0.00%)	.693
Effective (*n*)	23 (67.65%)	30 (78.95%)	25 (78.12%)	
Ineffective (*n*)	10 (29.41%)	7 (18.42%)	7 (21.88%)	
Total effective cases (*n*)	24 (70.59%)	31 (81.58%)	25 (78.12%)	

*Notes:* Total effective cases (*n*) = cured cases (*n*) + effective cases (*n*). Total effectiveness rate = [total effective cases (*n*)/total cases (*n*)] × 100%.

* *p* < .05 indicates statistically significant differences.

Fever resolution data were presented in Table 4. At baseline, the numbers of afebrile patients in the CRPA, CRAB and CRKP groups were 8 (23.53%), 8 (21.05%) and 9 (28.13%), respectively. After treatment, defervescence was attained in 17 (50.00%), 16 (42.11%) and 11 (34.37%) patients in the respective groups. Among these patients, the mean time to defervescence was 8.76 days for the CRPA group, 5.25 days for the CRAB group and 5.55 days for the CRKP group. There were no significant intergroup differences in the rates of fever resolution or the time required to achieve a normal temperature (*p* > .05, Table 4).

**Table 4.** Comparison of fever resolution among three groups of patients infected with different pathogens.

Outcome	CRPA (*n* = 34)	CRAB (*n* = 38)	CRKP (*n* = 32)	*p* Value
No fever (*n*)	8 (23.53%)	8 (21.05%)	9 (28.13%)	.718
Temperature normalized (*n*)	17 (50.00%)	16 (42.11%)	11 (34.37%)	
Temperature not normalized (*n*)	9 (26.47%)	14 (36.84%)	12 (37.50%)	
Temperature recheck time (days)	8.76 ± 5.54	5.25 ± 3.36	5.55 ± 3.53	.053

* *p* < .05 indicates statistically significant differences.

The CRGNB-attributable mortality rates for the CRPA, CRAB and CRKP groups were 35.29%, 21.05% and 34.38%, respectively, with no significant intergroup variations (*p* > .05). Similarly, the 28‑day survival rates for the CRPA, CRAB and CRKP groups were 52.94%, 65.79% and 43.75%, respectively, with no statistically significant difference among the three groups (*p* > .05) (Table 5 and Figure 2).

**Figure 2.**
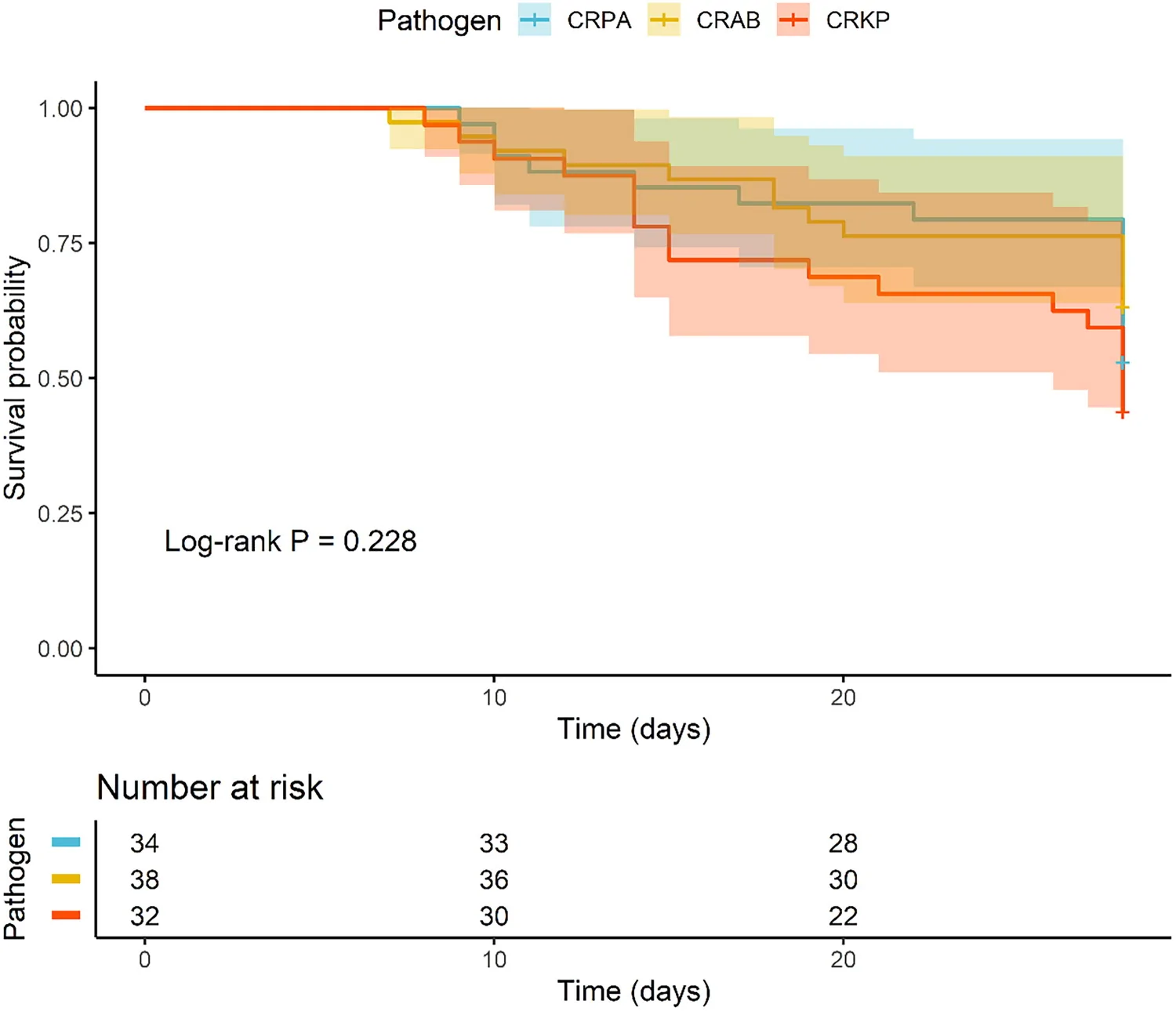
28-Day survival curves of patients infected with different pathogens.

**Table 5.** Comparison of causes of death and 28-day survival rate among three groups of patients infected with different pathogens.

Outcome	CRPA (*n* = 34)	CRAB (*n* = 38)	CRKP (*n* = 32)	*p* Value
CRGNB-related mortality (*n*)	12 (35.29%)	8 (21.05%)	11 (34.38%)	.333
28-Day survival (*n*)	18 (52.94%)	25 (65.79%)	14 (43.75%)	.228

*Notes: p* Values for CRGNB-related mortality were calculated using the Pearson χ^2^ test. The *p* value for 28-day survival was calculated using the log-rank test based on Kaplan–Meier survival analysis. *p* < .05 was considered statistically significant.

No significant changes in serum PCT levels were observed following PMB therapy across the three groups (*p* > .05). In contrast, serum CRP levels significantly declined after treatment compared with baseline in all three groups (all *p* < .05). For IL-6, a significant reduction after treatment was observed only in the CRAB group (*p* < .05), whereas IL-6 levels remained unchanged in the CRPA and CRKP groups (*p* > .05, Table 6).

**Table 6.** Comparison of inflammation marker levels among three groups of patients infected with different pathogens.

Item	CRPA (*n* = 34)	CRAB (*n* = 38)	CRKP (*n* = 32)
PCT (ng/mL)			
Before treatment	0.59 (0.20, 2.19)	0.73 (0.23, 3.40)	1.20 (0.22, 2.33)
After treatment	0.72 (0.19, 1.44)	0.85 (0.17, 2.99)	0.51 (0.13, 1.87)
*p* Value	.927	.880	.182
IL-6 (pg/mL)			
Before treatment	95.65 (30.15, 202.55)	76.40 (29.33, 144.90)	133.20 (59.98, 244.50)
After treatment	53.15 (16.88, 97.63)	38.60 (20.90, 79.25)	74.90 (35.73, 149.38)
*p* Value	.059	.033*	.157
CRP (mg/L)			
Before treatment	61.36 (32.79, 115.68)	87.98(46.71, 151.15)	114.55 (57.70, 193.42)
After treatment	38.10 (10.27, 69.69)	38.89(20.80, 96.24)	52.49 (21.48, 117.33)
*p* Value	.038*	.007*	.007*

* *p* < .05 indicates statistically significant differences.

No statistically significant intergroup differences were observed in the incidence of bronchospasm, nephrotoxicity, or skin hyperpigmentation during PMB therapy (all *p* > .05, Table 7). The safety profile was comparable across the CRPA, CRAB and CRKP groups.

**Table 7.** Comparison of the incidence of adverse reactions during treatment among three groups of patients infected with different pathogens.

Outcome	CRPA (*n* = 34)	CRAB (*n* = 38)	CRKP (*n* = 32)	*p* Value
Bronchospasm (*n*)	2(5.88%)	2(5.26%)	3(9.38%)	.769
Nephrotoxicity (*n*)	3(8.82%)	6(15.79%)	2(6.25%)	.399
Skin pigmentation (*n*)	1(2.94%)	4(10.53%)	3(9.38%)	.441

* *p* < .05 indicates statistically significant differences.

### 3.3. Factors associated with favourable bacterial clearance outcomes

All participants were divided into two groups based on microbiological status: the clearance group and the non-clearance group. Intergroup comparisons demonstrated that both *C*_peak_ and *C*_trough_ of PMB in ELF were significantly higher in the clearance group than in the non-clearance group (*p* < .001, Figure 3).

**Figure 3.**
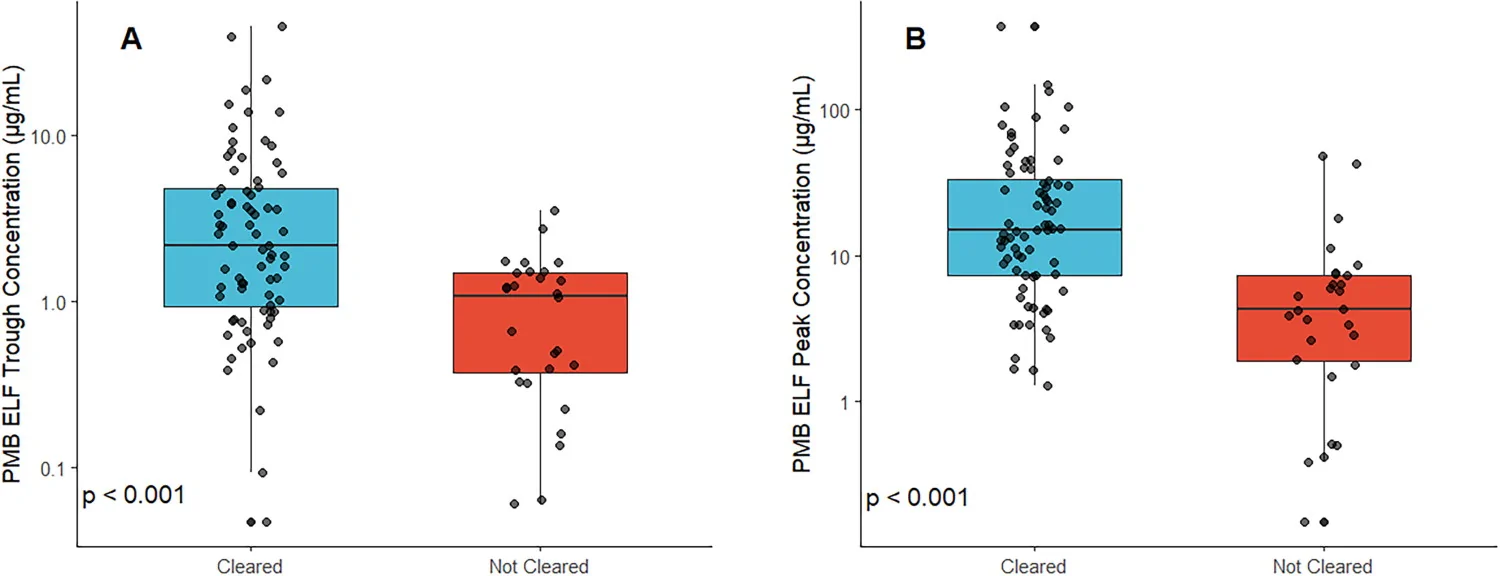
Comparison of the PMB ELF Concentration based on bacterial clearance.

Binary logistic regression was performed with bacterial clearance status as the dependent variable. The model included the following independent variables: PMB ELF *C*_peak_ and *C*_trough_, APACHE-II and SOFA scores, corticosteroid administration, duration of mechanical ventilation, duration of PMB therapy, pathogen type, PMB MIC and mean PMB plasma concentration. The results showed that PMB ELF trough concentration and duration of mechanical ventilation were independent predictors of bacterial clearance. Higher PMB ELF trough levels and longer duration of mechanical ventilation (OR = 1.133, 95%CI 1.033–1.242) were significantly associated with an increased likelihood of successful bacterial clearance (*p* < .05, Figure 4). Because of the limited number of non-clearance events, additional diagnostic and sensitivity analyses were performed. No severe multicollinearity was observed, and Firth’s penalized logistic regression yielded results consistent with the primary logistic regression model. These results are presented in Supplementary Tables S1 and S2.

**Figure 4.**
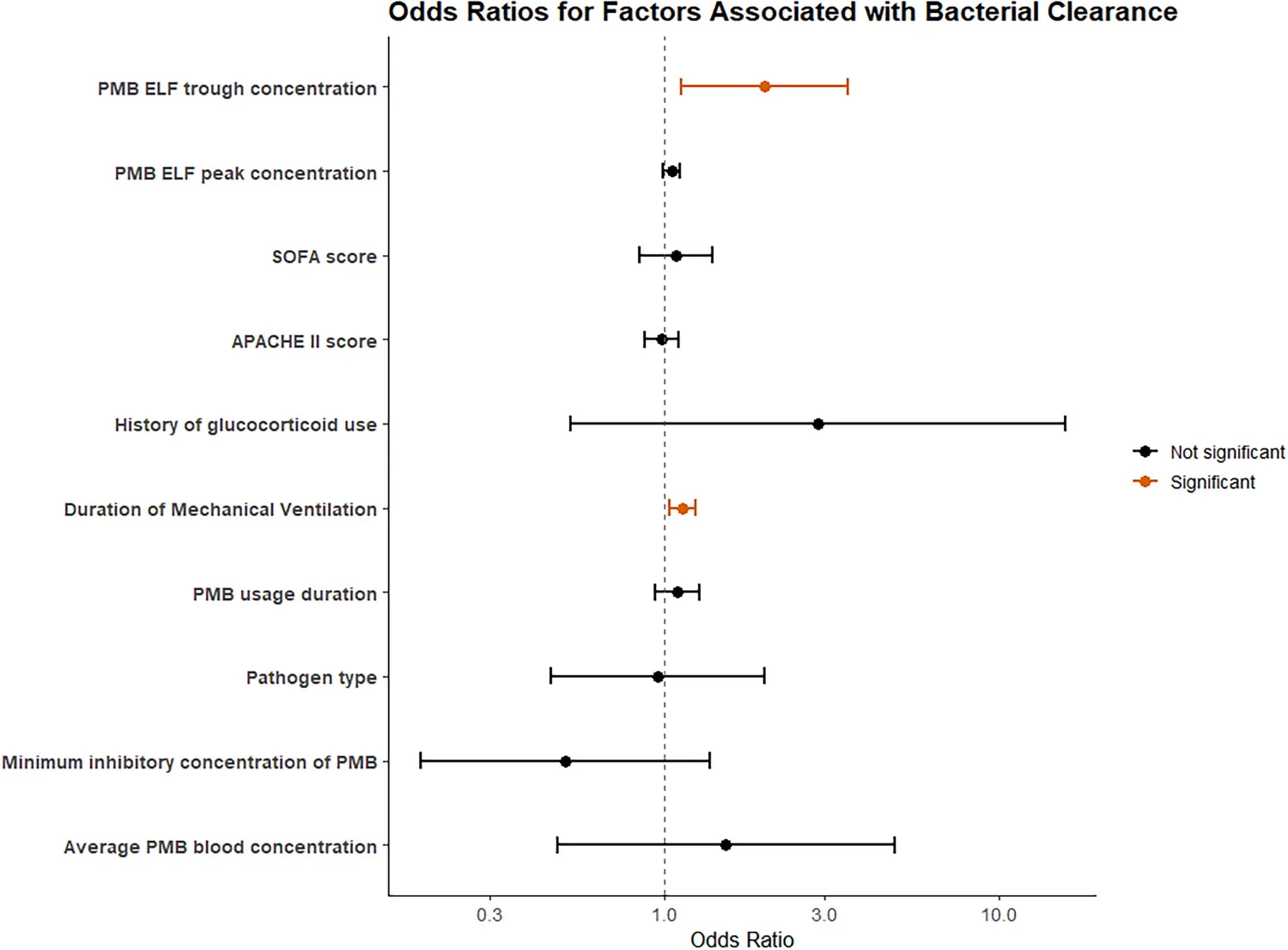
Forest plot of odds ratios (ORs) and 95% confidence intervals (CIs) for factors associated with bacterial clearance identified by binary logistic regression. Each point represents the estimated OR, with horizontal lines indicating 95% CIs. The dashed vertical line indicates the null value (OR = 1). Variables with *p* < .05 are shown in orange; non‑significant variables are shown in black.

### 3.4. Multiple linear regression analysis for predicting clearance/replacement time

Multiple linear regression analysis showed that PMB ELF C_peak_ was inversely associated with bacterial clearance/replacement time. This association remained significant after adjustment for age, sex, SOFA score, APACHE II score, corticosteroid use, duration of mechanical ventilation, PMB treatment duration, pathogen type, baseline MIC and mean PMB plasma concentration (Model 3: β = −0.241, *p* = .045). Similarly, PMB ELF *C*_trough_ was inversely associated with bacterial clearance/replacement time after full adjustment (Model 3: β = −0.239, *p* = .049) (Table 8).

**Table 8.** Multiple linear regression analysis of PMB ELF concentrations and bacterial clearance/replacement time.

	Model 1	Model 2	Model 3
PMB ELF C_peak_			
Beta	−0.271	−0.279	−0.241
*p* Value	.023	.021	.045
PMB ELF C_trough_			
Beta	−0.266	−0.285	−0.239
*p* Value	.024	.019	.049

*Notes:* Notes on multivariate linear regression models: Model 1, adjusted for age and sex. Model 2, adjusted for age, sex, SOFA score, APACHE II score, concomitant corticosteroid use and duration of mechanical ventilation; Model 3, adjusted for all variables in Model 2, with the addition of PMB treatment duration, pathogen type, baseline MIC and mean PMB plasma concentration.

* *p* < .05 indicates statistically significant differences.

## 4. Discussion

The relationship between PMB concentrations in ELF and the clearance of various pathogens remains poorly defined, which may compromise therapeutic efficacy. To address this gap, we conducted a prospective cohort study to compare bacterial clearance, clinical outcomes and adverse reactions among patients with pneumonia caused by different CRGNB strains who received combined nebulized and intravenous PMB. Additionally, we analysed the correlation between PMB ELF levels and microbial eradication to further elucidate its impact on clinical outcomes. Our findings demonstrate that combined nebulized and intravenous PMB is highly effective for CRGNB pneumonia. Notably, this regimen exhibited potent antimicrobial activity and a favourable safety profile against the predominant CRGNB strains at our centre, including CRAB, CRKP and CRPA, suggesting its potential as a viable treatment option in clinical practice [25]. Overall treatment outcomes were broadly comparable among patients with CRPA, CRAB and CRKP pneumonia, including bacterial clearance, clinical efficacy, survival, mortality and safety. The main pathogen-related difference was the bacterial clearance/replacement time, which was significantly longer in the CRAB group than in the CRKP group. Furthermore, while serum IL-6 levels significantly decreased after PMB treatment in the CRAB group, no significant changes were observed in patients with CRPA or CRKP infections. Higher C_peak_ and C_trough_ of PMB in ELF were associated with shorter time to bacterial clearance and higher eradication rates. Moreover, PMB ELF C_trough_ and duration of mechanical ventilation were identified as independent predictors of bacterial clearance. In this context, while high C_peak_ drive rapid initial bacterial reduction, maintaining sufficient C_trough_ levels is critical to prevent bacterial regrowth between doses, while longer duration of mechanical ventilation may reflect enhanced airway management, contributing to improved clearance outcomes.

Although PMB sensitivity, concomitant antibiotic regimens and diabetes prevalence differed among the three pathogen groups, no statistically significant differences in bacterial clearance rates, clinical efficacy rates, or CRGNB-related mortality were observed. The higher prevalence of diabetes in the CRPA group was unlikely to result from intentional selection, as patients were consecutively screened without selective or quota sampling. This imbalance may partly reflect the reported association between diabetes and *P. aeruginosa* infection in respiratory or other clinical infections [43,44]. Nevertheless, diabetes remains a potential confounder and should be considered when interpreting the results. Some patients received corticosteroid therapy. However, corticosteroid use did not differ significantly among the pathogen groups and was adjusted for in the multivariable models. Corticosteroids were unlikely to substantially affect PMB concentrations in ELF because pulmonary PMB exposure after nebulization is mainly determined by aerosol delivery and local lung conditions rather than corticosteroid-related metabolic pathways [5,8,30]. Nevertheless, a potential residual effect of corticosteroid therapy cannot be completely excluded. No statistically significant differences were observed among the three groups regarding total bacterial eradication rates, clinical success rates, or CRGNB-related mortality, all of which demonstrated favourable clinical outcomes. This consistency is likely attributable to the high PMB concentrations achieved in ELF after nebulization, particularly the C_peak_ which substantially exceeded the MICs. In the present study, ELF PMB concentrations represented the combined exposure after nebulized plus intravenous administration, and the separate contribution of each route could not be quantified. However, our previous pharmacokinetic studies suggested that nebulized PMB is the main driver of increased ELF exposure, whereas intravenous PMB mainly provides systemic exposure and contributes less to pulmonary ELF concentrations [21]. Given that the ELF serves as the predominant niche for extracellular pathogens during pneumonia, assessing localized drug exposure within this compartment provides a significantly more accurate reflection of therapeutic efficacy than systemic serum levels [45,46]. High concentrations of PMB at the target site achieved through nebulization exert potent antibacterial effects. Consequently, despite variations in MIC values across different pathogens, achieving elevated localized drug concentrations in the lungs ensures that the target ratio of free-drug area under the concentration–time curve to MIC (*f*AUC/MIC) is attained, thereby facilitating effective bacterial eradication [47].

However, our findings indicated that the time to bacterial clearance for CRAB was significantly longer than that for CRKP. This difference may be partly explained by pathogen-specific variations in PMB time-kill kinetics and PK/PD exposure requirements, as previous PK/PD studies have suggested that the PMB exposure needed to achieve comparable bacterial killing may differ across gram-negative pathogens [21]. Accordingly, CRAB may require higher local concentrations or longer exposure durations than CRKP to achieve similar bacterial eradication, despite comparable ELF concentrations. Additional mechanisms, such as biofilm formation, efflux pump activity and adaptive stress responses, may also contribute to delayed clearance, although these were not directly evaluated in the present study [48,49]. While post-treatment declines in CRP suggested clinical success in all cohorts, the discordant response of IL-6 which only decreased significantly in CRAB cases warrants further interpretation. By contrast, PCT levels showed no significant decrease after PMB treatment in any pathogen group, possibly reflecting persistent inflammatory stimulation by bacterial components such as lipopolysaccharide after bacterial killing, together with the relatively slow kinetic response of PCT in critically ill patients. PCT levels may also be influenced by organ dysfunction, trauma, surgery, systemic inflammation, or other non-infectious factors in ICU patients. CRAB appears to be a robust trigger for IL-6 production; thus, bacterial killing directly halts the inflammatory cascade at its source [50]. In contrast, the extensive tissue damage associated with CRPA and CRKP infections may act as a secondary stimulus for macrophage-mediated IL-6 release, even after microbial eradication, resulting in a less pronounced reduction of this cytokine [51].

Of note, our results revealed that both higher PMB ELF trough concentrations and longer durations of mechanical ventilation were associated with greater bacterial clearance. One possible explanation is that mechanical ventilation may theoretically facilitate aerosol delivery by generating controlled airflow and positive airway pressure, thereby potentially improving distal pulmonary deposition of nebulized PMB [52,53]. However, in our dataset, ventilation duration was not directly correlated with PMB ELF concentrations. Therefore, this association should be interpreted cautiously and regarded as hypothesis-generating rather than confirmatory. Reverse causation and survivor bias cannot be excluded; patients with slower bacterial clearance may require longer ventilatory support and those who survive longer may have more opportunity to receive PMB treatment and achieve microbiological clearance. Consequently, steady-state ELF C_trough_ are augmented, fulfilling the stringent pharmacokinetic/pharmacodynamic (PK/PD) requirements (sustained higher *f*AUC/MIC) necessary for effective bactericidal activity. This optimized pharmacometrics profile explains the more pronounced bacterial clearance observed in patients requiring extended ventilatory support [21]. These findings suggest that for patients with CRPA, CRAB, or CRKP pneumonia receiving nebulized PMB, optimizing aerosolization techniques and ventilatory modes to maximize pulmonary deposition may be beneficial. Such optimizations may facilitate the rapid attainment of effective high ELF C_trough_, thereby accelerating the control of severe infections [53]. Mechanistically, the duration of mechanical ventilation serves as a process-related variable and a facilitating factor for the attainment and maintenance of high ELF C_trough_. In contrast, elevated ELF C_trough_ represents the direct pharmacological driver responsible for potent bactericidal activity [5]. Together, these two factors provide a multidimensional explanation for the greater bacterial clearance observed in certain patient groups.

Further analysis revealed that the negative correlation between PMB ELF C_peak_ and C_trough_ and bacterial clearance time remained robust after adjusting for confounding factors. This indicates that both the initial loading intensity (C_peak_) and the sustained maintenance level (C_trough_) are independent and critical drivers of rapid bacterial clearance. Thus, an optimal eradication strategy requires a dual approach: ensuring a high initial C_peak_ for rapid bactericidal action, which further reinforces the concentration-dependent killing characteristics of PMB [54]. A sufficiently high initial C_peak_ is crucial for rapidly reducing the bacterial load. Subsequently, the maintenance phase must ensure sustained suppression through adequate C_trough_ to prevent bacterial regrowth and exert continuous selective pressure. This underscores that elevated C_trough_ are a critical component in maintaining the high *f*AUC/MIC ratios necessary for optimal clinical outcomes [21]. When the MIC of the infecting pathogen is high or approaches the breakpoint, maintaining adequate C_trough_ is paramount to ensure that the *f*AUC/MIC ratio exceeds the therapeutic threshold throughout the entire dosing interval. In cases of rapidly growing bacteria or in immunocompromised hosts, even if the initial C_peak_ eradicates the majority of the microbial population, suboptimal trough levels may allow residual bacteria to undergo rapid regrowth [55]. Elevated C_trough_ provide sustained antimicrobial pressure, which effectively inhibits bacterial regrowth and subsequently accelerates the overall eradication process [47]. These findings suggest the potential value of monitoring PMB exposure at the pulmonary target site. However, this study was not designed to establish definitive ELF concentration thresholds, predictive cut-off values, or therapeutic ranges. Future PK/PD studies are needed to define clinically actionable exposure targets, such as ELF C_peak_/MIC, C_trough_/MIC, or *f*AUC/MIC, for optimizing PMB dosing in CRGNB pneumonia.

Persistently low PMB concentrations in ELF may indicate insufficient target-site exposure and could increase the risk of treatment failure or selection of resistant subpopulations. Suboptimal pulmonary exposure may also contribute to heteroresistance, although this was not directly evaluated in the present study [5,28]. Therefore, enhancing PMB concentrations in ELF holds significant clinical implications. Firstly, higher ELF concentrations facilitate increased drug binding to lipid A on the bacterial outer membrane, disrupting membrane integrity and leading to leakage of cytoplasmic contents, which is vital for eradicating recalcitrant CRGNB [10]. This is particularly important for pathogens with elevated MICs, as many CRGNB (especially CRPA) exhibit PMB MIC values at the borderline of susceptibility breakpoints (e.g. 1–2 mg/L), necessitating elevated local concentrations to achieve target *f*AUC/MIC ratios for effective eradication [5,47]. Furthermore, in chronic pulmonary infections, CRGNB frequently form biofilms [49], which act as a formidable penetration barrier; high PMB concentrations are essential to penetrate the biofilm matrix and eliminate internal persister cells [26,56]. In addition to enhancing efficacy, achieving adequate ELF concentrations helps prevent the emergence of resistance. By maintaining ELF concentrations well above the MIC, a lethal strike can be achieved, maximizing bacterial kill and preventing resistant clones from becoming dominant – a strategy aligned with established PK/PD principles [5]. Moreover, nebulized PMB offers the advantage of ensuring therapeutic ELF levels while minimizing systemic exposure, thereby reducing the risk of nephrotoxicity and neurotoxicity associated with high-dose intravenous administration, particularly in immunocompromised patients [19,20].

This study has several limitations. First, it was a single-centre, observational study with a relatively small sample size; therefore, the findings require further validation through large-scale, multicentre randomized controlled trials. Second, significant inter-individual variability in PMB ELF concentrations was observed, which may be attributed to delivery obstruction caused by mucus plugs [52,53], impaired deposition efficiency in areas of lung consolidation [30,53] and technical factors such as drug dilution during BAL or sample processing losses [37]. Third, although no statistically significant differences were observed among the three pathogen groups in several outcome measures, these findings should not be interpreted as evidence of clinical equivalence, as this study was neither designed nor powered as an equivalence or non-inferiority trial. In addition, residual confounding is possible because MIC distributions and concomitant antibiotic regimens differed among pathogen groups and were closely related to pathogen type. Although these factors were considered in the multivariable analyses, their independent effects could not be fully separated from pathogen type. Larger prospective studies with more standardized treatment protocols are needed to validate these findings.

## 5. Conclusion

In conclusion, combined nebulized and intravenous PMB is an effective treatment strategy for CRGNB pneumonia caused by CRPA, CRAB and CRKP. This regimen achieves favourable bacterial eradication and clinical outcomes without increasing adverse events. Higher PMB concentrations in ELF were associated with shorter time to bacterial clearance and improved eradication rates. By enabling precise measurement of local drug concentrations through bronchoscopic-guided sampling and the urea dilution method, this study provides a pharmacological basis for individualized dose optimization and supports the clinical utility of combined PMB administration.

## Supplementary Material

Supplementary_Table_S1_Firth_Logistic.docx

Supplementary_Table_S2_Model_Diagnostics.docx

## Data Availability

The datasets used and/or analysed during the current study are available from the corresponding author on reasonable request. All data generated during the project will be made freely available *via* the ResMan Research Manager (ChiCTR2100044087) and Union Hospital, Fujian Medical University’s Research Data Repository.
