# Supplementary material for: Efficacy of polymyxin B against different pathogens in carbapenem-resistant gram-negative bacterial pneumonia and its relationship with alveolar epithelial lining fluid concentration: a prospective cohort study
Source: Ann Med. 2026 Nov 4;58(1):2752633. doi: 10.1080/07853890.2026.2752633 (PMC13757926; doi:10.1080/07853890.2026.2752633)
Supplement: Supplementary file 1 [file IANN_A_2752633_SM1632.docx]

## Supplementary Table S2. Diagnostic assessment and internal validation of the multivariable logistic regression model.

| **Item** | **Result** |
| --- | --- |
| Sample size | 104 |
| Number of events | 28 |
| Number of predictors | 11 |
| Events per variable (EPV) | 2.55 |
| **Adjusted GVIF range** | **1.02–1.48** |
| **Hosmer-Lemeshow goodness-of-fit** | **χ² = 6.24, P = 0.620** |
| **Area under the ROC curve (AUC)** | **0.891 (95% CI 0.825–0.958)** |
| =Bootstrap optimism | 0.142 |
| Optimism-corrected AUC | 0.821 |
| Bootstrap calibration slope | 0.532 |
| Optimism-corrected R² | 0.324 |
