# Supplementary material for: Efficacy of polymyxin B against different pathogens in carbapenem-resistant gram-negative bacterial pneumonia and its relationship with alveolar epithelial lining fluid concentration: a prospective cohort study
Source: Ann Med. 2026 Nov 4;58(1):2752633. doi: 10.1080/07853890.2026.2752633 (PMC13757926; doi:10.1080/07853890.2026.2752633)
Supplement: Supplementary file 2 [file IANN_A_2752633_SM1633.docx]

## Supplementary Table S1. Sensitivity analysis using Firth's penalized logistic regression.

| **Variable** | **Logistic regression OR (95% CI)** | **P (Logistic)** | **Firth logistic regression OR (95% CI, Wald)** | **P (Firth)** |
| --- | --- | --- | --- | --- |
| PMB ELF peak concentration | 1.05 (1.00–1.13) | 0.096 | 1.04 (0.99–1.09) | 0.118 |
| PMB ELF trough concentration | 2.21 (1.33–4.73) | 0.014 | 1.74 (1.14–2.67) | 0.003 |
| SOFA score | 1.07 (0.82–1.39) | 0.630 | 1.04 (0.83–1.29) | 0.748 |
| APACHE II score | 0.97 (0.86–1.10) | 0.672 | 0.98 (0.89–1.09) | 0.767 |
| History of glucocorticoid use | 2.84 (0.52–18.57) | 0.244 | 2.18 (0.50–9.60) | 0.321 |
| Duration of mechanical ventilation | 1.16 (1.06–1.30) | 0.003 | 1.13 (1.04–1.22) | 0.002 |
| PMB usage duration | 1.07 (0.91–1.26) | 0.429 | 1.05 (0.93–1.20) | 0.470 |
| Pathogen type (CRAB vs CRPA) | 0.25 (0.05–1.14) | 0.085 | 0.33 (0.09–1.26) | 0.108 |
| Pathogen type (CRKP vs CRPA) | 0.98 (0.20–4.70) | 0.985 | 0.99 (0.27–3.68) | 0.987 |
| Minimum inhibitory concentration of PMB | 0.35 (0.11–1.01) | 0.061 | 0.42 (0.17–1.02) | 0.077 |
| Average PMB blood concentration | 1.80 (0.54–6.72) | 0.355 | 1.57 (0.57–4.34) | 0.406 |

Note: Wald confidence intervals are presented for the Firth penalized logistic regression because profile penalized likelihood confidence intervals were unstable for some parameters. Results from Firth logistic regression are presented as sensitivity analyses to evaluate the robustness of the primary logistic regression model.
